# Two cases of posterior spinal fixation with titanium mesh cage and fenestrated pedicle screw for pyogenic spondylitis

**DOI:** 10.1093/jscr/rjaf122

**Published:** 2025-03-09

**Authors:** Soei Asuka, Keishi Maruo, Masaru Hatano, Shoji Nishio, Yoshiteru Nakamura, Toshiya Tachibana

**Affiliations:** Department of Orthopedic Surgery, Daiwa Central Hospital, Osaka City 557-0025, Japan; Department of Orthopedic Surgery, Hyogo Medical University, Nishinomiya City 663-8501, Japan; Department of Orthopedic Surgery, Hyogo Medical University, Nishinomiya City 663-8501, Japan; Department of Orthopedic Surgery, Daiwa Central Hospital, Osaka City 557-0025, Japan; Department of Orthopedic Surgery, Daiwa Central Hospital, Osaka City 557-0025, Japan; Department of Orthopedic Surgery, Hyogo Medical University, Nishinomiya City 663-8501, Japan

**Keywords:** pyogenic spondylitis, osteoporosis, cement augmented pedicle screw, titanium mesh cage

## Abstract

Pyogenic spondylitis in older patients with osteoporosis presents significant challenges due to implant failure and comorbidities. This study reports two cases of osteoporotic pyogenic spondylitis with substantial bony destruction, treated with cement-augmented pedicle screws (CAPS) and titanium mesh cages (TMC). Both patients achieved complete eradication of infection, spinal stabilization, and favorable clinical outcomes without recurrence or implant failure during follow-up. Patient 1 underwent posterior percutaneous pedicle screw fixation combined with CAPS and interbody fusion using a TMC via a costovertebral approach, whereas Patient 2 involved posterior vertebral body resection with TMC and CAPS to prevent cage subsidence. CAPS enhanced screw fixation and minimized complications related to poor bone quality. To our knowledge, this is the first report on the use of CAPS in the treatment off pyogenic spondylitis. The combined use of CAPS and TMC may offer a promising strategy for managing osteoporotic pyogenic spondylitis with extensive bony destruction.

## Introduction

The incidence of pyogenic spondylitis has been rising, especially among older adults [1, 2]. This is a growing concern owing to the increase in the older population and the prevalence of comorbid conditions in this population. Treatment options for pyogenic spondylitis in older adults include both conservative and surgical approaches. Surgical intervention is recommended for patients with spinal instability, severe pain, epidural abscesses, or neurological deficits [3, 4]. Percutaneous pedicle screw (PPS) fixation, a minimally invasive technique increasingly employed for spinal stabilization [5–7], has shown advantages over open surgery as it reduces operative time, blood loss, and postoperative complications while providing comparable infection control and mechanical stability [8]. However, PPS fixation in older patients is susceptible to screw loosening and implant failure and particular attention must be given to osteoporosis-related complications when using PPS in older patients.

Anterior reconstruction with a titanium mesh cage (TMC) is advocated for treating the treatment of spondylodiscitis with significant bony destruction. [5, 9]. Cement-augmented pedicle screws (CAPS) are increasingly used in spine surgery to enhance screw fixation, especially in patients with poor bone quality [10, 11]. In this report, we present two cases of pyogenic spondylitis in older patients with osteoporosis, successfully treated with CAPS and TMC, resulting in favorable surgical outcomes.

## Case report

### Case 1

**Figure 1:**
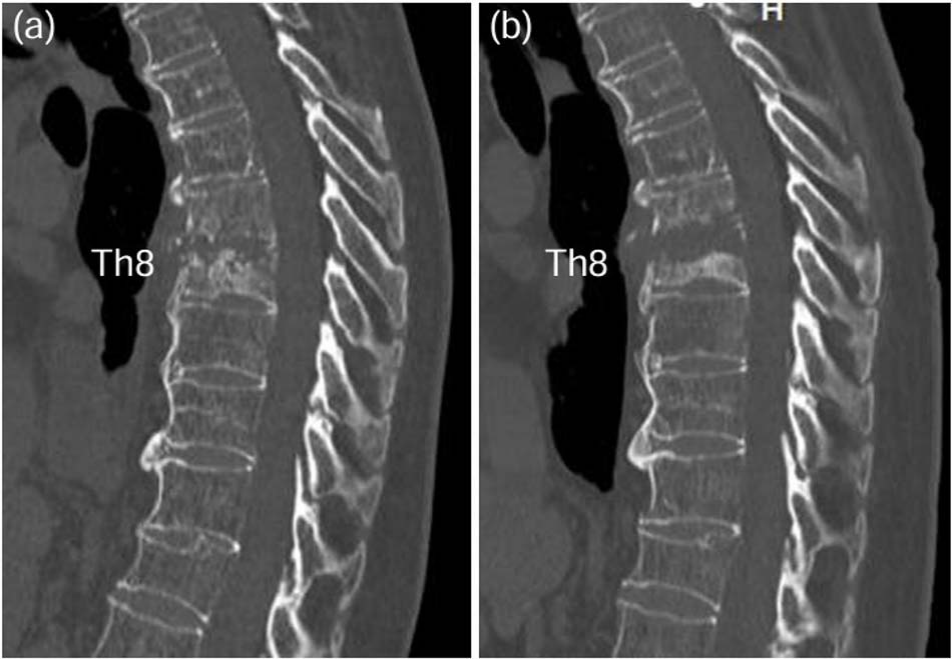
CT scan revealed osteolysis of the Th7-Th8 vertebral endplates and DISH upon admission (a). Four weeks later, progression of destructive changes at the Th7-Th8 endplates was observed (b).

**Figure 2:**
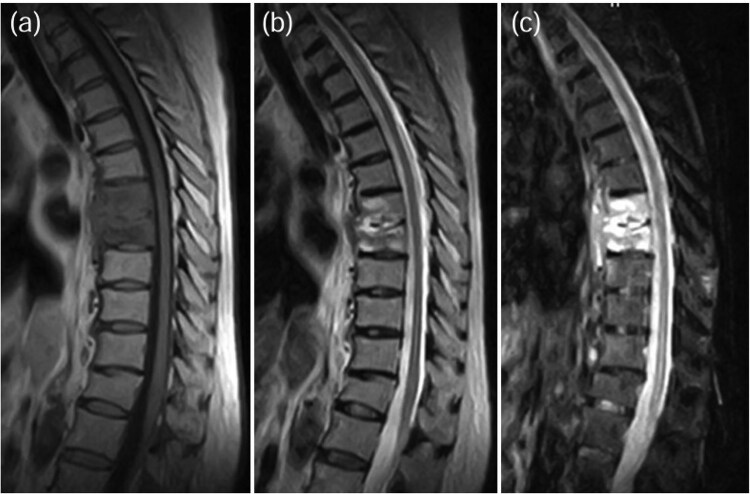
MRI demonstrated signal changes at Th7-Th8, with low intensity on T1-weighted images (a), high intensity on T2-weighted images (b), and no suppression on short-tau inversion recovery (STIR) images (c).

**Figure 3:**
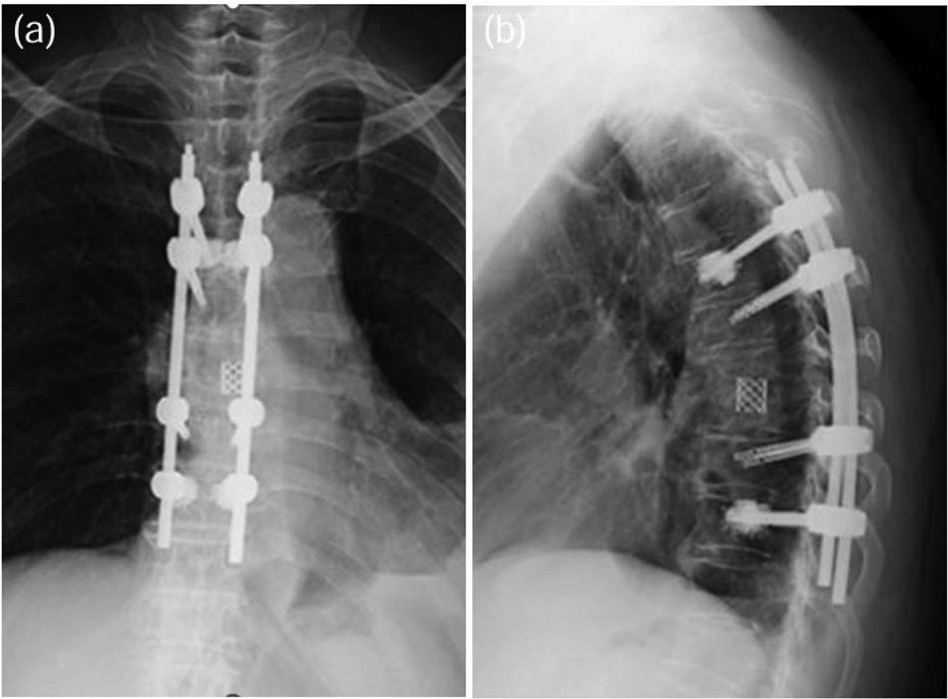
TMC replacement was performed at Th7-Th8, along with posterior fixation from Th5 to Th10 using CAPS at Th5 and Th10 [anterior–posterior view: (a), lateral view: (b)].

A 75-year-old woman presented with a 1-month history of lower back pain. She had no prior history suggestive of being immunocompromised. No neurological deficits were observed; however, the patient experienced severe back pain, which made it difficult to maintain a seated position. A computed tomography (CT)scan revealed erosion of showed the Th7-Th8 vertebral endplates, with associated diffuse idiopathic skeletal hyperostosis (DISH) (Fig. 1a). Blood tests showed a mild increase in inflammatory markers (C-reactive protein [CRP]: 1.02 mg/dL, white blood cells [WBC]: 6.0 × 10^3^ per μL). Magnetic resonance imaging (MRI) demonstrated signal changes in the Th7-Th8 region (Fig. 2). A biopsy of the Th7-Th8 intervertebral disk confirmed the presence of methicillin-susceptible *Staphylococcus aureus* (MSSA). She was diagnosed with Th7-Th8 pyogenic spondylitis and initially treated conservatively with antibiotics therapy (cefazolin) and a rigid brace. However, after 4 weeks, her symptoms persisted, and a follow-up CT showed further progression of the destructive changes at the Th7-Th8 endplates (Fig. 1b). Her WBC was 6.4 × 10^3^ per μL, and CRP was 1.19 mg/ dL. Spinal instrumentation surgery was planned, and an assessment of osteoporosis was conducted. The T-scores at the lumbar spine and total hip were −1.4 and −0.9, respectively, and the Hounsfield unit (HU) value at L1 was 79. Posterior PPS fixation was performed from Th5 to Th10 using CAPS at Th5 and Th10, along with the placement of a TMC at Th7-Th8 via a costovertebral approach (Fig. 3). Blood examination results returned to within the normal range at 3 weeks after surgery. Two years after surgery, there was no recurrence of infection and no evidence of screw loosening. The local kyphosis angle was 11° immediately postoperatively and was maintained at 10° at the final follow-up.

### Case 2

**Figure 4:**
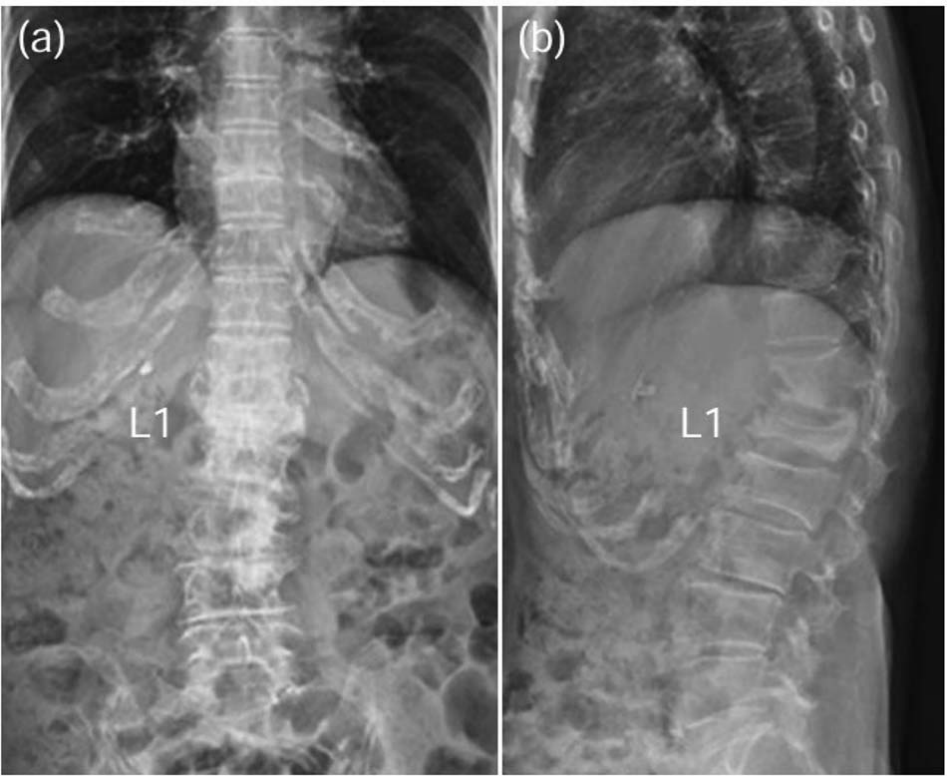
Plain radiographs obtained 6 months post-injury showed a cleft in the L1 vertebral body [anterior–posterior view: (a), lateral view: (b)].

**Figure 5:**
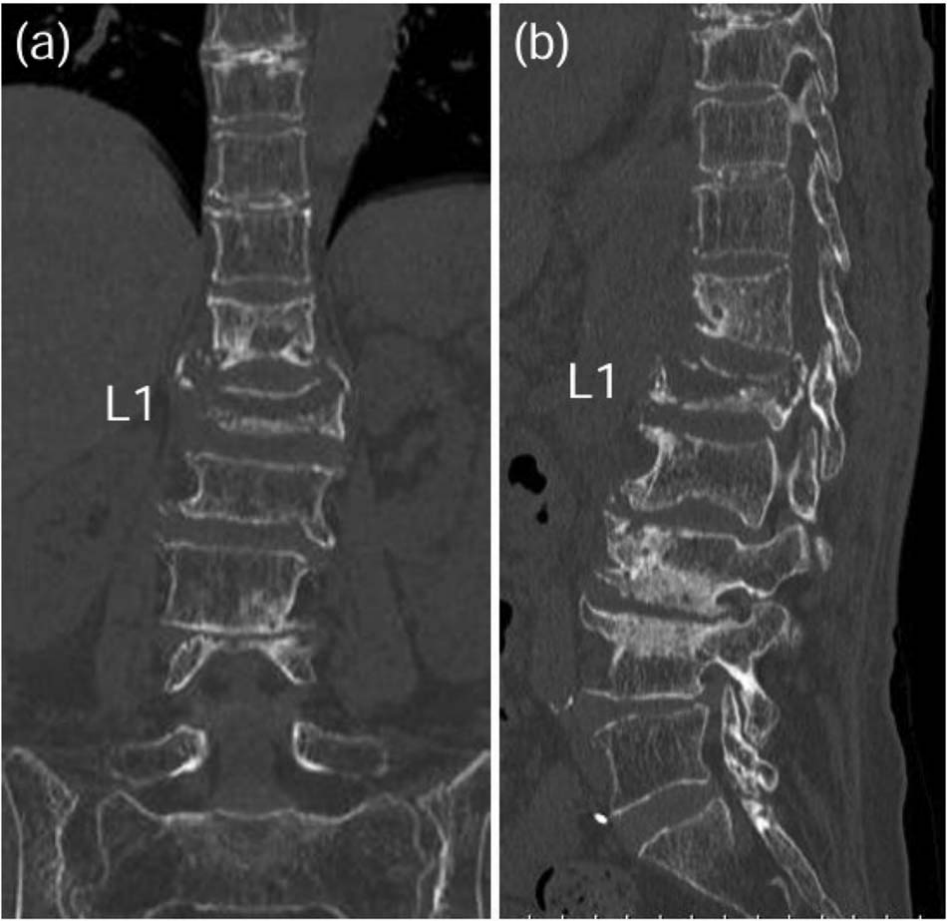
CT scan revealed osteolysis in the L1 vertebral body and the inferior endplate of Th12 [coronal view: (a), sagittal view: (b)].

**Figure 6:**
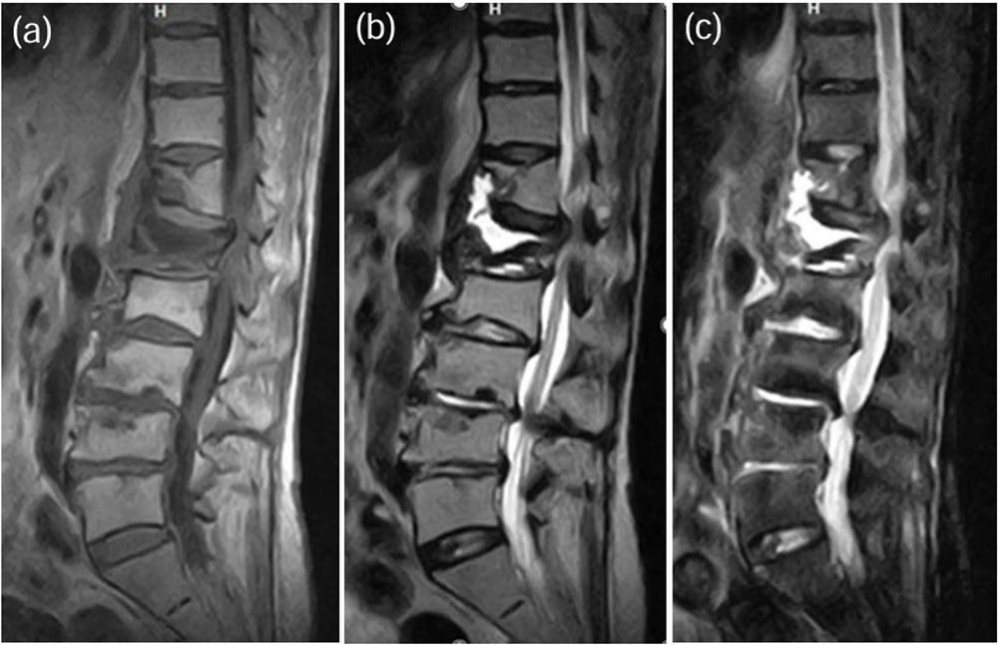
MRI demonstrated fluid signals in the L1 vertebral body and the anterior paravertebral area at Th12-L1. These were low-intensity on T1-weighted images (a) and high-intensity on T2-weighted images (b) and not suppressed on STIR images (c).

**Figure 7:**
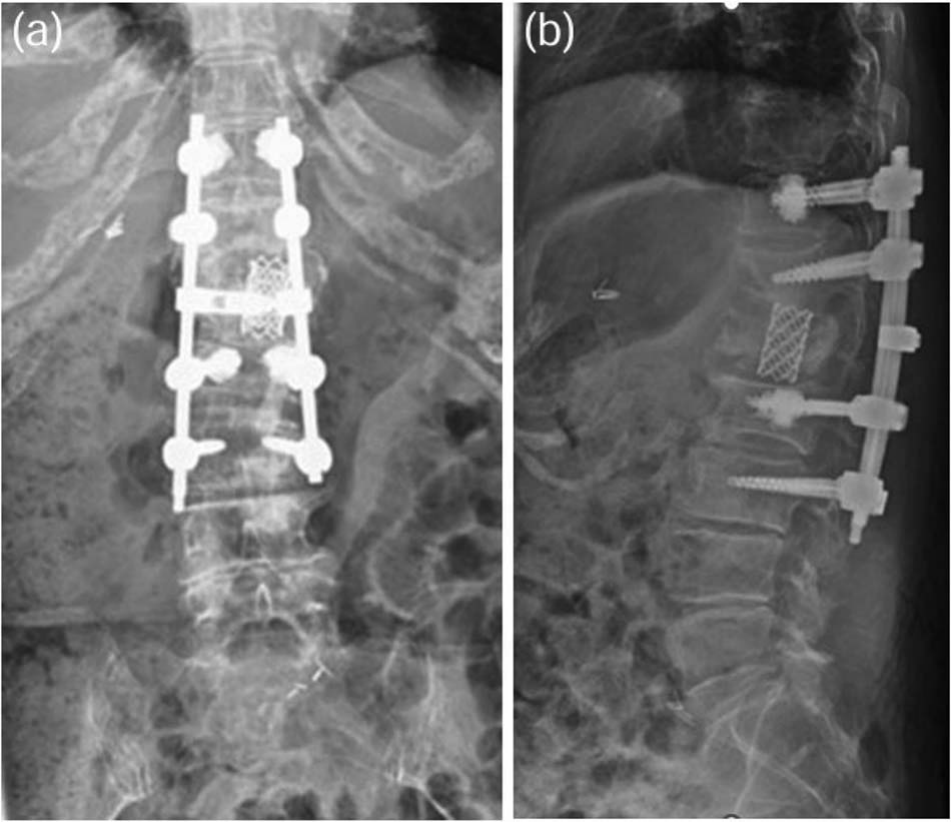
TMC replacement was performed at L1, with posterior fixation from Th11 to L3 using CAPS at Th11 and L2 [anterior–posterior view: (a), lateral view: (b)].

A 78-year-old woman was treated conservatively for an osteoporotic vertebral fracture at L1 with a rigid brace and osteoporosis medication. After 5 months of conservative treatment, her back pain persisted, and she subsequently developed lower limbs symptoms. She experienced severe thigh pain while standing. Plain radiographs showed an intervertebral cleft at L1 (Fig. 4), and a CT scan revealed osteolysis of the L1 vertebral body and the inferior endplate of Th12 (Fig. 5). MRI demonstrated high signal intensity changes in the L1 vertebral body and the anterior paravertebral area at L1-Th12 (Fig. 6). Blood tests showed a normal inflammatory response CRP: 0.28 mg/dL and WBC: 3.7 × 10^3^/μL). A biopsy of the L1 vertebral body revealed the presence of MSSA. The T-scores at the lumbar spine and total hip were −1.4 and −2.3, respectively. The HU value at L1 was 100. Posterior PPS fixation was performed using CAPS at Th11 and L2, along with the placement of a TMC at L1 via a posterior approach (Fig. 7). Eighteen months after surgery, there was no evidence of recurrent infection or screw loosening. The local kyphosis angle was 16° immediately postoperatively and was maintained at 15° at the final follow-up.

## Discussion

This study reports two cases of osteoporotic pyogenic spondylitis with substantial bony destruction treated successfully with CAPS and TMC. Both patients achieved complete eradication of infection, spinal stabilization, and favorable clinical outcomes without recurrence or implant failure during follow-up. Bettag *et al.* [12] highlight that the prevalence of osteoporosis in patients with pyogenic spondylitis is significantly underdiagnosed. The need for revision surgery resulting from implant failure may be associated with a estimated low HU value. Previous reports suggest that a HU value ˂110 is indicative of osteoporosis [12, 13]. In this case series, both patients exhibited low HU values; however, despite a normal T-score on dual-energy X-ray absorptiometry in Case 1, the low HU value led to a diagnosis of osteoporosis.

CAPS increases pullout strength and reduce the incidence of screw loosening, even in patients with osteoporosis [10, 11]. Recent studies have also demonstrated the safety and favorable surgical outcomes of hardware placement and pedicle screw insertion in infected vertebrae for the treatment of infectious spondylitis [14, 15]. However, no studies have reported the safety of using CAPS in infected vertebrae. Gamada *et al.* [15] reported that posterior PPS fixation for pyogenic spondylitis required unplanned reoperation in 24% of cases due to implant failure or reinfection. Our treatment strategy involves aggressive debridement to eradicate infection and achieve spinal stabilization, combined with the use of TMC in cases of significant bony destruction. In Case 1, we used CAPS at both the upper instrumented and lower instrumented vertebra (LIV). In Case 2, posterior vertebral body resection was performed with TMC, and CAPS was utilized at LIV-2 to prevent cage subsidence. No recurrence of infection or loss of correction due to screw loosening was observed.

To the best of our knowledge, there have been no reports on the use of CAPS for treating pyogenic spondylitis. The combined use of CAPS and TMC may offer an effective treatment option for osteoporotic pyogenic spondylitis associated with significant bony destruction. Further studies are needed to evaluate strategies for minimizing the extent of fusion and to assess long-term outcomes.
